# Circulating Matrix Metalloproteinases for Prediction of Aortic Dilatation in Children with Bicuspid Aortic Valve: A Single-Center, Observational Study

**DOI:** 10.3390/ijms251910538

**Published:** 2024-09-30

**Authors:** Amalia Făgărășan, Maria Oana Săsăran, Liliana Gozar, Daniela Toma, Carmen Șuteu, Simina Ghiragosian-Rusu, Flavia Cristina Al-Akel, Boglarka Szabo, Adina Huțanu

**Affiliations:** 1 Department of Pediatrics III, Faculty of Medicine, George Emil Palade University of Medicine, Pharmacy, Science and Technology of Târgu Mureș, 540142 Târgu Mureș, Romania; amalia.fagarasan@umfst.ro (A.F.); liliana.gozar@umfst.ro (L.G.); daniela.toma@umfst.ro (D.T.); carmen.suteu@umfst.ro (C.Ș.); simina.ghiragosian-rusu@umfst.ro (S.G.-R.); 2 Department of Pediatric Cardiology, Emergency Institute for Cardiovascular Diseases and Transplantation of Târgu Mureș, Gheorghe Marinescu Street No 50, 540136 Târgu Mureș, Romania; alakelcristina@gmail.com (F.C.A.-A.); szabo.boglarka9512@gmail.com (B.S.); 3 Department of Pediatrics III, Faculty of Medicine in English, George Emil Palade University of Medicine, Pharmacy, Sciences and Technology of Târgu Mureș, Gheorghe Marinescu Street No 38, 540142 Târgu Mureș, Romania; 4 Pathophysiology Department, Faculty of Medicine in English, George Emil Palade University of Medicine, Pharmacy, Sciences and Technology of Târgu Mureș, 540142 Târgu Mureș, Romania; 5 Department of Laboratory Medicine, Faculty of Medicine, George Emil Palade University of Medicine, Pharmacy, Science and Technology of Târgu Mureș, 540142 Târgu Mureș, Romania; adina.hutanu@umfst.ro

**Keywords:** bicuspid aortic valve, aortic dilatation, aortic aneurysm, matrix metalloproteinase, children

## Abstract

Circulating biomarkers have been proposed for early identification of aortic dilatation progression associated with bicuspid aortic valve (BAV), but matrix metalloproteinases (MMPs) are distinguished as signatures of increased extracellular matrix degradation, a landmark of aneurysm formation. The current study aims to identify the role of MMP-1, MMP-2, MMP-9, and the MMP inhibitor, TIMP-1, in identifying aortic dilation in children with BAV. We conducted a study on 73 children divided into two study groups, depending on the presence of aortic dilatation (group 1–43 BAV controls and group 2–30 children with BAV and aortic dilatation). Each patient underwent a cardiac ultrasound and, in each case, serum MMP-1, MMP-2, MMP-9, and TIMP-1 were quantified using xMAP technology. Comparison of the MMPs between the two study groups revealed significantly higher values only in the case of TIMP-1, among BAV controls. Moreover, the same TIMP-1 inversely correlated with aortic annulus absolute size and z score, as well as with ascending aorta z score. No particular correlation between the aortic phenotype and the presence of aortic dilatation was found. Future longitudinal research starting at pediatric ages could show the significance of MMPs screening in BAV individuals as predictors of aortic aneurysm formation.

## 1. Introduction

The progressive dilatation of the aorta occurs in a series of syndromic and non-syndromic congenital cardiac malformations (CCM). BAV has a prevalence of approximately 0.8% in children and is characterized by a great variety of clinical presentations [1]. Its diagnosis could be established even in the neonatal period, especially when associated with critical CCM [2,3,4,5].

The association between BAV and structural alterations of the aortic wall has been researched in detail, including the propensity of BAV patients towards developing thoracic artery aneurisms [6,7]. Studies emphasize the importance of genetic and hemodynamic factors in the development of aortic dilatation. The predominantly symmetric dilation of the aortic root starts at an early age, and aortopathy is a product of the hemodynamic turbulences, which are caused by the genetically determined, anatomically abnormal aortic valve. After birth, these will represent triggers and accelerating factors of the local degeneration of the aortic wall [8,9,10]. One study conducted on a population from Denmark reported a prevalence of aortic dilatation around 30% among newborns with BAV, without other concurring cardiac malformation [2].

The development of aortic dilatation seems to be dependent upon the extracellular matrix (ECM) degradation and remodeling. The active involvement of matrix metalloproteinases and tissular inhibitor of metalloproteinase contributes to the maintenance of the aortic wall homeostasis [11]. Fibrillin-1 is a fundamental extracellular matrix component of the aortic media and maintains the integrity and elasticity of the aortic wall [12]. Its underexpression has been associated with increased matrix degradation triggered by MMPs and with the development of aortic aneurysms [12]. In subjects with malformations of the aortic valve, medial degeneration has been reported, with underlying fibrillin-1-deficient tissue and increased MMP production [13,14]. Degeneration of the media, characterized by fragmentation of the elastic fibers and apoptosis of smooth muscular cells, is even more accelerated in patients with BAV and aortic stenosis [15,16,17].

The proven link between degeneration of the aortic media and BAV yielded the research of potential biomarkers of disease progression. MMPs seem to be involved in valve remodeling, with imbalance between MMPs and their inhibitors, tissue inhibitors of MMPs (TIMPs), such as an MMP-9/TIMP-1 showcasing a role in alteration of valvular morphology [18]. Moreover, MMPs are key regulators of ECM homeostasis and especially control this process through association with extracellular vesicles [19]. Some MMPs, such as MMP-3, might even be transported to target cells and afterwards exercise their action inside the cell [20]. There are also membrane-type matrix metalloproteinases (MT-MMPs), which are found in mesenchymal cells and trigger macrophage invasion and consequent ECM modeling, especially through activation of other MMPs [21,22,23]. Still, as extracellular-vesicles-associated MMPs seem to be long-distant influencers of ECM restructuring, these have more frequently been proposed as biomarkers of disease progression and are easier to quantify [24].

The thoracic aorta seems to be more sensitive to inadequate extracellular matrix synthesis [25]. Independent of aneurysm location, research for easily quantifiable, circulating biomarkers which can predict progression of aortic dilatation and aortic aneurysm dissection has included, among several serum biomarkers, MMPs as well, in light of their proven role in vascular wall remodeling [26]. Associations between MMPs and parameters such as wall shear stress (WSS) and aortic strain were also found [27]. In a similar fashion, other MMPs proved their potential biomarker role in prediction of evolving aneurysms of the abdominal aorta [28]. In particular, MMP-2, found in mesenchymal cells, has been linked to macrophage invasion and seems to be the elastase which has the highest tissular concentration in abdominal aortic aneurysms [22].

In BAV, experimental data have shown that genetic determinism of BAV further translates into predisposition towards aortic dilatation development. The influence of aortic valve morphology on MMP expression was studied and results showed that animals with BAV present a significant decrease in MMP-2 expression [29]. Although several candidate biomarkers in BAV have been further studied, due to young ages, pediatric data are distinguished through paucity [30]. Hence, the current study proposes to identify whether three MMPs, namely MMP-1, MMP-2, and MMP-9, and the MMP inhibitor, TIMP-1, could be used to predict aortic dilatation in children with BAV.

## 2. Results

The 73 children included in this study had a mean age of 12.63 ± 0.45 years, and male sex represented 75.34%. In terms of valvular phenotype, type 0 (without raphe) was found in 32.87% of the cases, type IA, IC, and II were only encountered in 6.84%, 4.1% of cases, and 2.73%, respectively, and 53.42% of children presented a IB phenotype. The study population was divided into two groups, dependent on the presence of aortic dilation. Therefore, group 2 (study group) consisted of 30 children with aortic dilation, whereas group 1 was comprised of 43 age-matched subjects with no evidence of aortic diameter modifications (control group). A comparison between demographic, anthropometric, and ultrasound features of the two groups is depicted through Table 1. In terms of gender distribution, a significantly higher prevalence of the female sex was noted in the control group, as opposed to group 2 (*p* < 0.01, OR = 0.2). No significant differences between the two study groups were found in terms of background, body mass index (BMI), or body surface. When analyzing ultrasound parameters of the aorta, significantly higher values for the aortic annulus (absolute value and z score), sinus (z score), junction (absolute value and z score), and for the ascending aorta diameter were measured for group 2 (*p* < 0.01). Ejection fraction (EF) and fractional shortening were similar between the two study groups, but a significantly higher mean left ventricular diameter was identified in those patients with aortic dilatation (4.54 ± 0.84 SD versus 4.12 ± 0.60 SD, *p* = 0.03). Nevertheless, there were no significant discrepancies in prevalence of particular aortic phenotypes between the two groups.

A possible influence of the particular aortic valvular phenotype on the development of aortic dilatation was onwards investigated. Chi square test was applied to assess differences in the prevalence of gender, aortic stenosis, and aortic stenosis degree between three main groups of aortic phenotypes (0, IB, and IA/IC/II). Given that only four patients from the entire study population did not present aortic insufficiency, phenotype-dependent prevalence of aortic insufficiency was not analyzed. Similar gender ratios were found, irrespective of aortic phenotype (*p* = 0.49). A significantly higher prevalence of phenotype IB was found among patients who did not present aortic stenosis (32.87%, *p* = 0.04). However, similar distribution of the three aortic phenotype groups was found in patients with mild, moderate, or severe aortic stenosis (*p* = 0.31). These results are represented in Table 2. When applying a logistic regression analysis, no association between the valvular phenotype and the presence of aortic dilation was found (*p* = 0.11). Aortic phenotype was also not significantly dependent upon gender (*p* = 0.06). Moreover, the valvular phenotype did not correlate with the presence of aortic stenosis (*p* = 0.36) nor with its severity (*p* = 0.13, *p* = 0.30, and *p* = 0.15, respectively). In a similar fashion, the valvular phenotype did not seem to influence the presence of aortic insufficiency (*p* = 0.74) nor its severity.

In order to evaluate potential gender-based variation of MMPs, we firstly compared circulating MMP values in female and male patients. As Table 3 shows, there was no significant gender-based discrepancy for any of the studied MMPs.

A comparison of NT-proBNP levels and circulating levels of the targeted MMPs between the two groups was also conducted. No significant discrepancies were found in NT-proBNP, MMP-1 or MMP-3, vitamin D nor C reactive protein (CRP) circulating levels. Still, TIMP-1 presented significantly higher mean values in the control group (57.87 ± 18.29 SD versus 49.84 ± 15.49 SD, *p* = 0.04). These results can be visualized in Table 4.

We further sought to conduct the same comparison of the potential circulating biomarkers in a study subgroup with aortic stenosis, divided in a similar fashion, based on the presence of aortic dilatation. Therefore, those 38 patients in whom an aortic stenosis was diagnosed were divided into group A (18 patients), which comprised subjects without aortic stenosis, and group B (20 patients), which included cases with aortic dilatation. No statistically significant variations were seen in any of the studied parameters (NT-proBNP, MMP-1, MMP-2, and MMP-3) between the two groups (Table 5).

**Figure 1:**
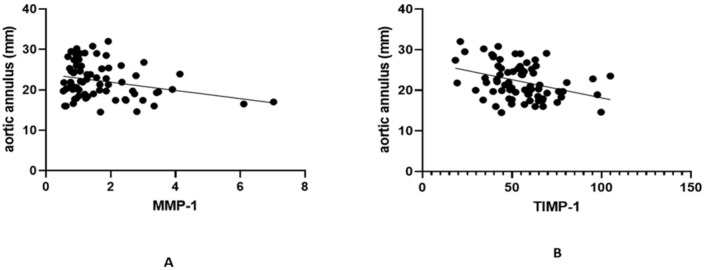
Correlation between absolute size of aortic annulus and serum biomarkers. (**A**) Correlation between absolute size of aortic annulus and serum MMP-1. (**B**) Correlation between absolute size of aortic annulus and serum TIMP-1.

**Figure 2:**
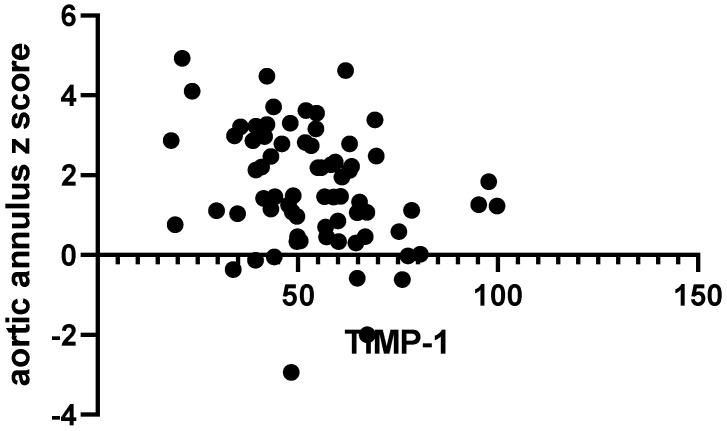
Correlation between absolute size of aortic annulus and serum TIMP-1.

We tried to assess how gender, BMI, and serum vitamin D levels influence values of MMPs and TIMP-1. Therefore, we performed multiple linear regression for each individual MMP studied but found that none of the confounders influenced their values when taken separately nor when analyzing two-way or three-way interactions. By computing the Spearman correlation, MMP values were individually investigated in relation to the presence of aortic dilatation. However, MMP-1, MMP-2, MMP-9, and TIMP-1 values obtained in our study were unpredictive of the presence of aortic dilation (*p* = 0.27, *p* = 0.11, *p* = 0.52, and *p* = 0.05, respectively). Correlations between the circulating serum biomarkers and the size of aortic annulus were further investigated. An inverse, significant correlation was found between MMP-1 and TIMP-1 levels and the absolute size of the aortic annulus (*r* = −0.27, *p* = 0.01 and r = −0.36, *p* < 0.01), as portrayed through Figure 1A,B. The same correlations between serum parameters and the z score of the aortic annulus were assessed, but only a unique, significant inverse association between TIMP-1 and aortic annulus z score was found (r = −0.28, *p* = 0.01, Figure 2).

**Figure 3:**
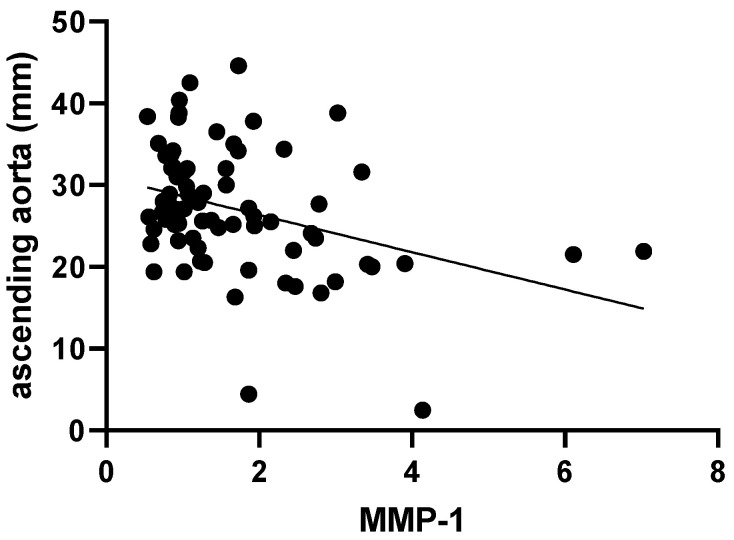
Correlation between absolute size of the ascending aorta and serum MMP-1.

**Figure 4:**
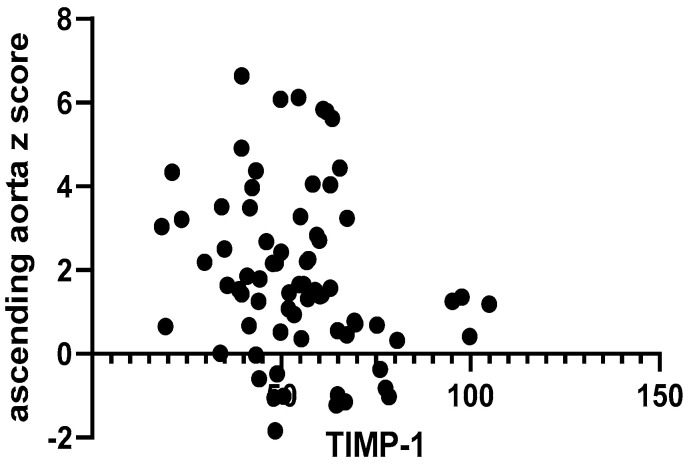
Correlation between ascending aorta z score and serum TIMP-1.

MMP-1 values were inversely related to the absolutely measured diameter of the ascending aorta (r = −0.35, *p* < 0.01, Figure 3), unlike the other investigated MMPs and TIMP-1, which showed no significant interrelation with the same parameter. However, when analyzing the same relationship between the aforementioned parameters and the z score of the ascending aorta, only TIMP-1 showed a significant correlation (r = −0.24, *p* = 0.03, Figure 4).

**Figure 5:**
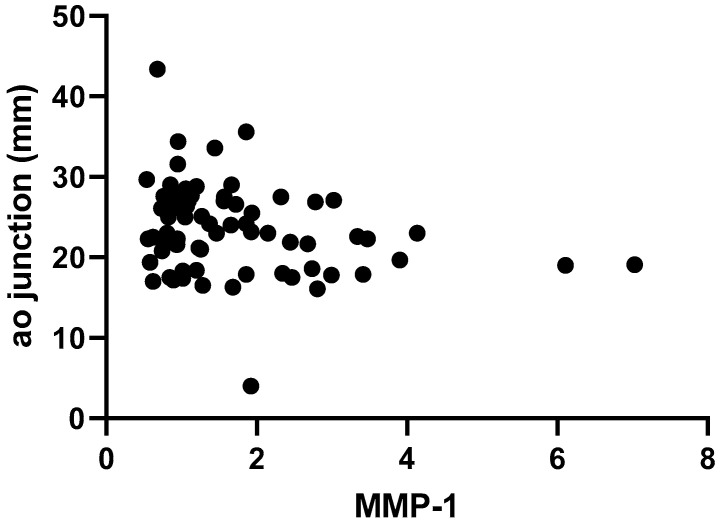
Correlation between absolute size of the aortic junction and serum MMP-1.

MMP and TIMP values did not correlate with aortic sinus measurements, as expressed in both mm and z scores. When studying the same relationship between MMP-1, MMP-2, MMP-9, TIMP-1, and the aortic junction, a unique significant correlation emerged, as MMP-1 increase was correlated with lower absolute values of the aortic junction (r = −0.26, *p* = 0.02, Figure 5).

## 3. Discussion

Besides an increased prevalence in the general population, BAV is an important contributing factor to pediatric morbidity due to co-existing valvulopathy–aortopathy complexes, which include aortic stenosis/insufficiency and/or aortic dilation, occurring at different childhood stages [1,5,8,31]. The extracellular matrix seems to play an essential role in ensuring optimal morpho-functional parameters of the aortic valvular cusps and of the aortic walls. Moreover, the balance between MMPs and their inhibitors, TIMPs, is crucial [32]. Furthermore, genetic factors have also been proven to be involved in the development and progression of aortic dilatation, as they represent contributing factors for extracellular matrix imbalance and for the premature apoptosis of vascular smooth muscle cells [9,16,33,34].

Besides the structural modifications of the aortic wall which might favor the onset of aortic dilation, the BAV phenotype seems to influence the development of this complication, although data are still controversial. Three-dimensional studies of the blood flow dynamic have shown that phenotype IA determines an asymmetric blood flow, which modifies the exterior curve of the proximal ascending aorta. In the case of IB phenotype, the flow is posteriorly redirected towards the proximal ascending aorta [34]. Still, studies have shown that the IB phenotype is most commonly associated with ascending aortic dilatation and it usually coexists with aortic insufficiency [35,36]. However, this phenotype is more frequently encountered in Asian populations than in Europeans [37]. Within our study, 41.09% percent of children with BAV presented an aortic dilatation and the majority of the patients presented the IB phenotype. No particular correlation was found between the aortic phenotype and aortic dilatation.

A corner stone in daily clinical practice is the establishment of a risk scale for critical events (aortic dissection) and to choose the optimal surgery timing in case of valvulopathy development (with/without aortic dilatation). Therefore, the identification of novel, easily quantifiable biomarkers (laboratory parameters or imagistic) for risk stratification is required, given that critical complications of aortic dilatation can be silent. Markers related to the molecular, architectural modifications of the vascular wall have been proposed for premature identification of aortic dilatation. MMPs were among the first enzymes described, with collagenolytic functions, in 1962 [38].

MMP-2 and MMP-9 remain the most intensely studied MMPs in relation to aneurisms. As a matter of fact, MMP-2 has been proven to detain the highest collagenolytic activity and is involved in the fragmentation of sarcomeric proteins contained within cardiac myocites, which is a result of lesions induced by oxidative stress [39,40]. Therefore, MMP-2 is involved in the modulation of inflammatory intracellular pathways and cardiac metabolism and has been quantified as a predictor of heart failure [41]. Other studies have shown that MMP-9 is the most expressed gelatinase in abdominal aortic aneurysm, especially when compared to the essential role of MMP-2 and MMP-9 in aneurysm production having been highlighted by an experimental study, which showed that deficiency of both MMPs in mice will protect against the production of abdominal aortic aneurysm [28]. Upregulation of both MMP-2 and MMP-9, as well as of MMP-1, has been reported in aneurysms of the abdominal aorta [18,42,43,44]. Increased proteolytic activity of MMP-2 and MMP-9 and consequent high circulating values of these MMPs have also been found in connection to abdominal aortic aneurysm rupture [45]. Yet, these studies analyzed tissular MMP changes in subjects who already had critical aortic dilatations. The quantification of serum MMPs and the intent of establishing their utility in the progression of aortic dilatation led to subsequent studies. Although fewer in number, some studies proved that circulating MMP-1, MMP-2, and MMP-9 present similar augmentation in thoracic aortic aneurysms, aortic dissection, can predict the necessity of surgery in subjects with aneurisms of the ascending aorta, and are even correlated with ultrasound parameters such as WSS and time-average WSS (TAWSS) [27,46,47]. The overexpression of MMPs is reversible, as proven by a study conducted on patients with abdominal aortic aneurysm, in whom two different surgical approaches yielded a similar decrease in MMP-3 and MMP-9 levels [48]. Persistent release in the bloodstream of MMP-9 has further been related to aortic degeneration, increase in aneurysm size, and its expansion. The same study proved that MMP-9 can be inhibited by doxycycline, which could represent a novel therapeutic measure [49]. Our pediatric study, however, did not find any significant difference in serum MMP-1, MMP-2, and MMP-9 levels between children with BAV and those with BAV and aortic dilatation. Comparison of the MMPs in patients with aortic stenosis, divided based on the presence of aortic dilatation, yielded similar results. Previous pediatric data analyzing the behavior of MMPs in childhood BAV are limited to one study, which found no significant differences in TIMP-1 and MMP-9 values between children with isolated BAV, without any underlying heart conditions, and those with tricuspid aortic valve [50].

TIMPs emerged as inhibitors of neoangiogenesis, which aim to prevent the degradation of the extracellular matrix and aneurysm formation. One study conducted on a population with thoracic aortic aneurysm who underwent surgery revealed that the aortic specimens of these individuals were abundant in MMP-1 and MMP-9 expression, whereas TIMP-1 and TIMP-2 were barely expressed, and were more easily detectable towards the outer layers of the vessel [51]. The increase in MMPs expression and the decrease in TIMPs, with the consequent disbalance of MMP/TIMP ratios will promote proteolysis, extracellular matrix degradation, and formation of abdominal aortic aneurysms, according to Tamarina et al. [42]. Within our study, we found significantly higher TIMP-1 values within the control group, which might suggest its protective role against aneurysm formation. There is still debate surrounding the role of TIMP-1 in establishing the risk of aortic dilatation. Tzemos et al., who focused on the variation in MMP-2, MMP-9, TIMP-1, and TIMP-2 in young men with BAV and dilation of the proximal aorta, reported a significant increase only in MMP-2 when comparing the study group with BAV controls with normal aortic diameter [52].

There are important limitations to our study as follows: it was conducted at a single center and it was conducted on a relatively small sample size. Another limitation of the current study would be the use of the z score for assessment of aortic dilatation. One study conducted on an Italian pediatric population with BAV suggested that a novel score, namely the Q score, based on a machine-learning algorithm, might be more accurate in evaluating the presence of aortic dilatation than the z score, which tends to exaggerate the incidence of aortic dilatation, especially among adolescents [53]. In spite of the setbacks, our study is distinguished through the enrolment of a pediatric population with BAV, in whom the role of various MMPs and TIMP-1 in the prediction of aortic dilatation was analyzed. Due to the unavailability of previous pediatric data, we cannot compare the measured values with age-dependent reference ranges, but the results obtained could represent a starting point for expansion of future pediatric research on the same subject.

## 4. Materials and Methods

### 4.1. Study Population

The study population consisted of 73 children aged between 6 and 17 years of age, enrolled over a period of one year (between 10 January 2023 and 10 January 2024), presenting for regular check-ups in a tertiary pediatric cardiology referral center, with previously known BAV, diagnosed through cardiac ultrasound. The study population was divided into two groups, based on the identification of aortic dilatation (group 1—BAV controls and group 2—subjects with BAV and aortopathy). Exclusion criteria consisted of genetic syndromes, metabolic disorders, fibrotic hepatic disease, renal or chronic respiratory illness, oncological diagnoses, as well as any type of condition that would imply prolonged steroid treatment, which is known to influence MMP serum levels.

### 4.2. Cardiac Ultrasound Evaluation

Every subject was examined with the help of a Philips-EPIQ CVx 3D nSIGHT Plus ultrasound machine (Philips, United States of America, Andover, Massachusetts). The acquired images were digitally stored and, later, offline revisualized and analyzed. In order to establish the aortic valve phenotype, two experienced pediatric cardiologists evaluated the parasternal short-axis view sections, and the valvular phenotype classification was conducted in accordance with the raphes number and the spatial configuration of the cusps. The standard currently available terminology was used, based on the most recently published data for BAV and associated aortopathies [54] Thus, the aortic bicuspid phenotypes were classified as follows: fused BAV (1A—right–left cusp fusion, 1B—right non-cusp fusion, 1C—left non-cusp fusion and indeterminate phenotypes); 2-sinus BAV (laterolateral and anteroposterior phenotypes); and partial-fusion BAV/or mild BAV forms (small raphe, single phenotype).

The parasternal long-axis view was used to calculate z scores, after proper image optimization for the aortic root and the proximal ascending aorta (Figure 1), perpendicular to the long aortic axis of the aorta, inner-edge technic (aortic annulus, aortic root, sinotubular junction—STJ, and ascending aorta—1 cm distal to the STJ) [54].

The value of the diameter in each case was obtained based on the mean of the diameters measured in three consecutive beats. Z scores were based on body surface area calculated by the Haycock formula (dilation of the aorta was diagnosed based on z scores > 2) and the Cantinotti method was applied [55].

The diagnosis of aortic stenosis was based upon the measurement of the peak and mean pressure gradients (with the help of continuous wave Doppler ultrasound), which were calculated through the Bernoulli simplified equation. Therefore, aortic stenosis was divided into mild—mean gradient < 20 mm Hg and peak velocity < 3 m/s, moderate—mean gradient ranging between 20–40 mm Hg, and severe—mean gradient > 40 mm Hg and valve peak velocity > 4 m/s. This classification is in accordance with the guideline recommendations [56]. The aortic valve regurgitation was divided into absent/none, medium, and severe. This division was based upon jet width relative to annular diameter ratio, assessed in the parasternal long-axis view, on the descending aortic backflow on color Doppler, left ventricular dimensions (mild—normal, moderate—normal or dilated, and severe—usually dilated) and Jet deceleration rate—CW (PHT pressure half-time, ms), slow < 500, medium 500–200, and severe < 200 [57].

Evaluation of the LV systolic function was conducted in compliance with the current guidelines for systolic function analysis; the EF was calculated using the Teicholz formula (in the absence of asynchrony signs) and the modified Simpson method (2D apical four chambers).

### 4.3. Serum Biomarker Quantification

For the quantification of peripheral MMP-1, MMP-2, MMP-9, and TIMP-1, venous blood samples were collected in the EDTA tubes, centrifuged, and plasma samples were aliquoted and stored at −80 °C until all patients were recruited. For laboratory measurement, the plasma samples underwent a dilution of 20-fold for the MMPs panel and 50-fold for TIMP-1. Afterwards, the protocol according to the manufacturer’s instructions was followed. Plasma levels of MMP-1, MMP-2, MMP-9, and TIMP-1 were assessed with Human MMP magnetic bead panel 2 (EMD Millipore Corp, Berlington, MA, USA) and Human TIMP Magnetic Bead panel 1, respectively, using xMAP technology (Luminex, DiaSorin S.p.A, Saluzzo, Italy).

Diluted plasma samples were incubated with analyte-specific monoclonal antibody-precoated magnetic beads. After defined incubations, reagents addition, and washing steps, antigen–antibodies sandwich complexes resulting at the end of the reaction were analyzed on the Flexmap 3D platform (Luminex, DiaSorin S.p.A, Saluzzo, Italy). After the fluorochrome addition (phycoerythrin), the complexes were interrogated by two lasers available on the equipment by a flow cytometry principle. The red laser interrogated the type of magnetic bead, and each bead category was classified based on the internally dyed color. In contrast, the green laser measured the concentration of each analyte, expressed as median fluorescence (MIF). The concentration of each analyte was estimated using a 5-parameter logistic curve, automatically generated by the software (xPONENT 4.2).

The measuring interval for MMP-1 ranged between 27 and 20,000 pg/mL with intra-assay coefficients of variation (CV) of 2.6% and 8.4% for inter-assay precision. For MMP-2 the measuring range was 68–50,000 pg/mL, with intra-assay CV of 5.4% and intra-assay CV of 18%. For MMP-9, the measuring interval was 14–10,000 pg/mL, with intra-assay CV of 1.9% and 9.0% for inter-assay. In the case of TIMP-1, the measuring interval was 20–20,000 pg/mL with CVs ≤ 10% for intra-assay and inter-assay precision. Values outside the measuring range were extrapolated by the xPONENT software 4.2.

### 4.4. Statistical Analysis

The entire statistical analysis was performed with the help of GraphPad Prism 9.0.2 software. A confidence interval of 95% was applied, which signified that only *p* values lower than 0.05 were considered statistically significant. Descriptive statistics involved calculation of variables such as mean, frequency, and standard deviation. The distribution of quantitative data was evaluated using the Kolmogorov–Smirnov normality test. Mean comparison was conducted with the help of the unpaired *t* test with Welch’s correction for Gaussian distributed variables and by applying the Mann–Whitney test for those variables without a Gaussian distribution. In case of categorical/binary variables, Chi square test was applied. For establishing potential correlations between cytokine values and the presence of aortic dilatation and aortic ultrasound parameters, Spearman and Pearson correlations were computed. Multiple linear regression was used to identify how possible confounders might influence MMP values.

### 4.5. Ethics

The entire research followed the principles stated in the declaration of Helsinki. Prior to inclusion in the study, at least a legal tutor of each of the patients enrolled signed an informed consent form and agreed to the subjects’ study enrollment, cardiac ultrasound assessment, and blood withdrawal for biomarker quantification. The research protocol was approved by the Ethics Committee of the Târgu Mureș Emergency Institute for Cardiovascular Diseases and Transplantation (approval no. 8902/20.12.2022) and by that of the George Emil Palade University of Medicine, Pharmacy, Science, and Technology of Târgu Mureș (approval no. 2034/26.01.2023).

## 5. Conclusions

Several factors can influence the development of aortic dilatation in subjects with BAV, such as the particularity of the flow direction, exerted upon the aortic wall, determined by the aortic phenotype. As the involvement of peculiar morphological valvular subtypes has been controversial, novel biomarkers of extracellular matrix cleavage have been proposed, with possible determination through noninvasive methods, in conjunction with close echocardiographic monitoring. Our study, conducted on a pediatric population with BAV, found no particular connection between the valvular phenotype and the development of aortic dilatation and revealed no significant discrepancies in circulating MMP-1, MMP-2, or MMP-9 values between BAV controls and BAV subjects presenting with dilatation of the aorta. As the control group exhibited significantly higher TIMP-1 values, this result suggests the potential protective effect of TIMP-1 against extracellular matrix degradation and the need for early screening of its level in BAV populations. Still, the small sample size and the unicentric enrolment of the study subjects limits the power of the study. Hence, further longitudinal studies are required to assess the importance of MMP screening in BAV individuals for prediction of aortic aneurysm formation. These will need to periodically assess in-dynamic changes of MMPs, especially in relation to other well-established cardiac biomarkers, and to enroll wider population samples from different geographic areas.

## Figures and Tables

**Table 1:** Comparison of demographic, anthropometric, and echocardiographic data between the two study groups.

Parameter		Group 1 (n = 43)	Group 2 (n = 30)	*p* Value
Age (mean ± SD)		12.01 ± 3.54	13.42 ± 4.11	0.07
Gender	Male (%)	38.35	36.98	*p* = 0.01, OR = 0.2
	Female (%)	20.54	4.10	
Background	Urban (%)	35.61	26.02	*p* = 0.80, OR = 0.88
	Rural (%)	23.28	15.06	
BMI (mean ± SD)		19.43 ± 4.49	20.02 ± 4.06	0.39
Body surface (mean ± SD) *		1.44 ± 0.40	1.54 ± 0.40	0.30
Aortic stenosis (n = 38)	Mild (%)	21.05	36.84	0.03
	Moderate (%)	18.42	2.63	
	Severe (%)	7.89	13.15	
Aortic insufficiency (n = 61)	Mild (%)	52.45	29.50	0.12
	Moderate (%)	6.55	8.19	
	Severe (%)	0	3.27	
Aortic annulus (mm, mean ± SD)		21.09 ± 3.97	23.83 ± 4.42	<0.01
Aortic annulus (z score, mean ± SD)		1.26 ± 1.10	2.28 ± 1.72	<0.01
Aortic sinus (mm, mean ± SD)		24.82 ± 4.90	26.84 ± 8.18	0.23
Aortic sinus (z score, mean ± SD)		0.01 ± 1.43	1.24 ± 1.87	<0.01
Aortic junction (mm, mean ± SD)		22.01 ± 4.11	25.95 ± 6.87	<0.01
Aortic junction (z score, mean ± SD)		1.11 ± 1.05	2.81 ± 1.56	<0.01
Ascending aorta (mm, mean ± SD)		24.26 ± 5.95	31.09 ± 8.09	<0.01
Ascending aorta (z score, mean ± SD)		0.83 ± 1.23	3.36 ± 1.94	<0.01
EF (%, mean ± SD)		66.63 ± 7.78	68.17 ± 8.17	0.42
Left ventricle diameter (mean ± SD)		4.12 ± 0.60	4.54 ± 0.84	0.03
Fractional shortening (%, mean ± SD)		38.16 ± 7.71	38.52 ± 7.34	0.84
Aortic phenotype	0 (%)	17.80	15.06	0.61
	IB (%)	34.24	19.17	
	IA/IC/II (%)	6.84	6.84	

Legend: BMI—body mass index; EF—ejection fraction; SD—standard deviation. *—*t* Student test was used.

**Table 2:** Distribution of aortic phenotype in relation to gender, the presence of aortic stenosis, and degree of aortic stenosis.

Variable		Aortic Phenotype			*p* Value
		0 (n = 22)	IB (n = 39)	IA/IC/II (n = 12)	
Gender	Female (%)	8.21	15.06	1.36	0.49
	Male (%)	24.65	38.35	12.32	
Aortic stenosis	Yes (%)	20.54	20.54	10.95	0.04
	No (%)	9.58	32.87	5.47	
		0 (n = 17)	IB (n = 15)	IA/IC/II (n = 6)	
Aortic stenosis	Mild (%)	21.05	23.68	13.15	0.31
	Moderate (%)	7.89	10.52	2.63	
	Severe (%)	17.14	5.26	0	

**Table 3:** Serum level comparison of NT-proBNP, MMPs, and TIMP-1 between female and male study subjects.

Parameter	Female Patients (n = 18)	Male Patients (n = 55)	*p* Value
MMP-1 (pg/mL, mean ± SD)	1.79 ± 1.74	1.73 ± 1.15	0.42
MMP-2 (pg/mL, mean ± SD)	143.1 ± 20.62	142.5 ± 30.31	0.49
MMP-9 (pg/mL, mean ± SD)	117.9 ± 52.21	128.2 ± 74.95	0.92
TIMP-1 * (pg/mL, mean ± SD)	57.53 ± 15.75	53.60 ± 18.12	0.41

Legend: *—*t* Student test was used.

**Table 4:** Serum level comparison of NT-proBNP, MMPs, and TIMP-1 between the two study groups.

Parameter	Group 1 (n = 43)	Group 2 (n = 30)	*p* Value
NT-pro-BNP (ng/mL, mean ± SD)	22.51 ± 19.16	39.66 ± 53.58	0.37
MMP-1 (pg/mL, mean ± SD)	1.86 ± 1.39	1.43 ± 0.78	0.27
MMP-2 (pg/mL, mean ± SD)	144.6 ± 24.23	139.9 ± 33.11	0.11
MMP-9 (pg/mL, mean ± SD)	132.5 ± 78.54	115.8 ± 54.83	0.31
TIMP-1 * (pg/mL, mean ± SD)	57.87 ± 18.29	49.84 ± 15.49	0.04
Vitamin D (ng/L, mean ± SD)	39.57 ± 21.89	39.29 ± 24.07	0.93
CRP (mg/dL, mean ± SD)	0.34 ± 0.20	0.42 ± 0.22	0.28

Legend: CRP—C reactive protein; MMP—matrix metalloproteinase; NT-proBNP—N-terminal pro-B-type natriuretic peptide; SD—standard deviation; TIMP—tissue inhibitor of metalloproteinase. *—*t* Student test was used.

**Table 5:** Serum level comparison of NT-proBNP, MMPs, and TIMP-1 in subjects with aortic stenosis and normal aortic diameter/aortic dilatation.

Parameter	Group A (n = 18)	Group B (n = 20)	*p* Value
NT-pro-BNP (ng/mL, mean ± SD)	14.87 ± 13.75	33.54 ± 54.44	0.22
MMP-1 (pg/mL, mean ± SD)	2.06 ± 1.84	1.50 ± 0.89	0.61
MMP-2 (pg/mL, mean ± SD)	144.1 ± 19.05	140 ± 37.97	0.15
MMP-9 (pg/mL, mean ± SD)	139.4 ± 73.78	109.1 ± 52.93	0.23
TIMP-1 * (pg/mL, mean ± SD)	53.90 ± 14.24	49.28 ± 13.73	0.31
Vitamin D (ng/L, mean ± SD)	42.25 ± 21.99	38.97 ± 22.92	0.87
CRP (mg/dL, mean ± SD)	0.28 ± 0.16	0.31 ± 0.24	0.48

Legend: MMP—matrix metalloproteinase; NT-proBNP—N-terminal pro-B-type natriuretic peptide; TIMP—tissue inhibitor of metalloproteinase. *—*t* Student test was used.

## Data Availability

The original contributions presented in the study are included in the article; further inquiries can be directed to the corresponding author.
